# Insights into the Molecular Epidemiology of Foot-and-Mouth Disease Virus in Russia, Kazakhstan, and Mongolia in Terms of O/ME-SA/Ind-2001e Sublineage Expansion

**DOI:** 10.3390/v15030598

**Published:** 2023-02-21

**Authors:** Viktor Nikiforov, Alexey Shcherbakov, Ilya Chvala, Svetlana Kremenchugskaya, Fedor Korennoy, Tamara Mayorova, Anna Timina, Samat Tyulegenov, Sarsenbay Abdrakhmanov, Maksat Berdikulov, Tserenchimed Sainnokhoi, Delgerzul Gombo-Ochir, Tsagaan Tserenchimed, Larisa Prokhvatilova, Alexander Sprygin

**Affiliations:** 1 Federal Center for Animal Health, Vladimir 600901, Russia; 2 S. Seifullin Kazakh Agrotechnical University, Astana 010000, Kazakhstan; 3 RSE National Veterinary Reference Center, Astana 010000, Kazakhstan; 4 State Central Veterinary Laboratory, Ulaanbaatar 17024, Mongolia

**Keywords:** foot-and-mouth disease, epidemiology, evolution, VP1

## Abstract

Foot-and-mouth disease (FMD) has long been recognized as a highly contagious, transboundary disease of livestock incurring substantial losses and burdens to animal production and trade across Africa, the Middle East, and Asia. Due to the recent emergence of the O/ME-SA/Ind-2001 lineage globally contributing to the expansion of FMD, molecular epidemiological investigations help in tracing the evolution of foot-and-mouth disease virus (FMDV) across endemic and newly affected regions. In this work, our phylogenetic analysis reveals that the recent FMDV incursions in Russia, Mongolia, and Kazakhstan in 2021–2022 were due to the virus belonging to the O/ME-SA/Ind-2001e sublineage, belonging to the cluster from Cambodian FMDV isolates. The studied isolates varied by 1.0–4.0% at the VP1 nucleotide level. Vaccine matching tests indicated that the vaccination policy in the subregion should be tailored according to the peculiarities of the ongoing epidemiologic situation. The current vaccination should change from such vaccine strains as O_1_ Manisa (ME–SA), O no 2102/Zabaikalsky/2010 (O/ME-SA/Mya-98) (r_1_ = 0.05–0.28) to strains that most closely antigenically match the dominant lineage O No. 2212/Primorsky/2014 (O O/ME-SA//Mya-98) and O No. 2311/Zabaikalsky/2016 (O ME-SA/Ind-2001) (r_1_ = 0.66–1.0).

## 1. Introduction

Foot-and-mouth disease (FMD) is a highly contagious viral disease affecting domestic and wild cloven-hoofed animals manifesting with the development of vesicles, chiefly in the mouth and on the feet coupled with a fever [1]. The causative agent belongs to the Picornaviridae family and the *Aphthovirus* genus [2]. The viral genome is a single-stranded positive-sense RNA of approximately 8 kb in length, containing a single open reading frame encoding four structural proteins and eight nonstructural proteins (NSPs). The viral capsid comprises structural proteins VP1, VP2, VP3, and VP4, while the other NSPs are mainly involved in viral replication and pathogenesis [3]. The VP1 protein acts as the main immunogenic component of the FMDV virion [4].

FMD causes limited mortality and severe morbidity levels in infected herds, disrupting regional and international trade in animals and animal products [5,6]. The World Organization for Animal Health has included FMDV on its list of notifiable diseases due to severe impacts and significant production losses by FMDV [7]. Over 70% of the world’s cattle population is affected by FMD, and it has attained endemic status in Africa, the Middle East, Asia, and limited areas of South America.

FMDV exists as seven immunologically distinct serotypes based on VP1: A, O, Asia-1, SAT-1, SAT-2, SAT-3, C with a diversity of topotypes, genetic lineages, and strains [8]. Within endemic regions, FMDV serotypes are not evenly distributed, with serotype O having the widest distribution [1]. There is no cross-protection between the different serotypes [9,10]; thus, infection or vaccination with one FMDV serotype does not protect against others [11].

The phylogenetic analysis of the VP1 genomic region identified 10 topotypes of the FMDV O serotype that occur in East Africa (EA 1-4), West Africa (WA), Europe–South America (EURO-SA), Indonesia (ISA 1-2), Middle East–South Asia (ME–SA), and Southeast Asia (SEA) [12]. In Southeast Asia, there are three cocirculating topotypes: the O/SEA/Mya-98 lineage, the CATHAY topotype, and the Middle East–South Asia (ME–SA) topotype [13,14,15]. However, a novel lineage, O/ME-SA/Ind-2001, appeared in India in 2001 [16] and has recently raised concerns due to its range expansions coupled with ongoing multiple transregional movements. Since 2001, it spread to Saudi Arabia and the United Arab Emirates in 2013 [17], Bahrain in 2015 [18], and North African countries, including Libya in 2013 [19], Tunisia and Algeria in 2014 [17], and Morocco in 2015 [20], Southeast and East Asia [21,22], and Cambodia and Pakistan in 2018 [23,24]. The alarming rate of expansion may lead to high risks of causing a pandemic and further spillover into regions up north.

The Russian Federation and the Republic of Kazakhstan are nonendemic nations, with FMD-free zones recognized by the OIE. In previous years, sporadic FMD cases were reported in Russia that were attributed to spillovers from infected domestic and wild cloven-hoofed animals of neighboring countries. The isolates were found in 2014, 2016, 2018, 2019, and 2020 in the region of Zabaikalsky Krai of Russia, which shares its borders with China and Mongolia, and belonged to the O serotype, prevalent in China and Mongolia, and sporadic cases of the A serotype were detected in 2013 [25].

Interestingly, sporadic cases in Kazakhstan in 2007 and 2010 were caused by O/ME-SA/PanAsia2 isolates that had been transmitted from Central Asia. However, from 2011 to 2013, genetic lineages of O/ME-SA/PanAsia and A/Asia/Sea-97 circulating in China and SEA escaped and extended their range into Kazakhstan [26,27]. Following the emergence of O/ME-SA/PanAsia strains that derived from South Asia, this lineage rapidly spread and attained panzootic status [28].

In 2021–2022, FMD outbreaks were reported in Mongolia, Kazakhstan, and Russia (collectively referred to as subregions), which share borders with countries with an active circulation of O/ME-SA/Ind-2001 lineage strains, revealing the unprecedented transboundary and aggressive movement of FMD in a northward direction [29].

This work aims to describe and characterize the genetic and antigenic relationships of FMDV isolates from Russia, Kazakhstan, and Mongolia to gain insights into the molecular evolution of FMDV strains together with circulating strains from FMD-affected neighboring countries in the subregion.

## 2. Materials and Methods

### 2.1. Sampling and Control Measure Taken during the Outbreak

In this study, we analyzed samples from three countries with shared borders: the Russian Federation, Kazakhstan, and Mongolia.

**Figure 1:**
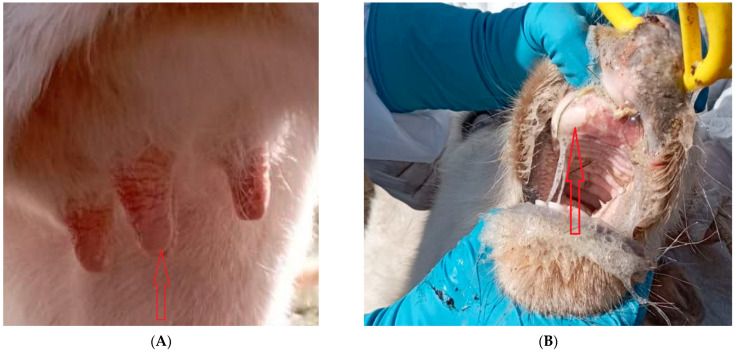
Clinical signs observed at a foot-and-mouth disease outbreak identified in Orenburg oblast in December 2021. (**A**) lesions on the treats, (**B**) aphts in the mouth.

In the Russian Federation, the FMD outbreak occurred in the village of Karagach, Belyaevskii rayon, Orenburg region in a backyard unit with 17 cattle and 22 sheep. A total of 2 cows were affected and exhibited clinical signs (Figure 1A,B).

**Figure 2:**
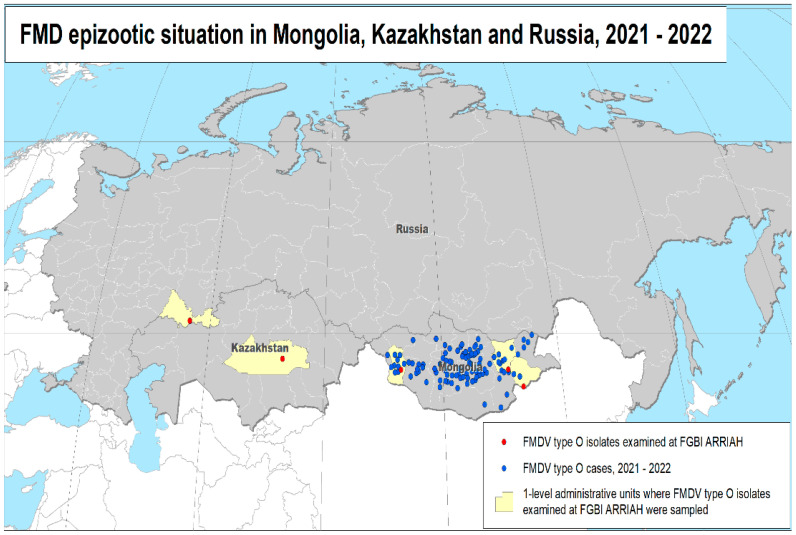
Map showing foot-and-mouth disease outbreaks in 2021 and 2022 in the subregion.

In December 2021, two biological samples (aphthous ulcers) were collected from FMD-infected cattle (from two cows owned by one owner) in the Orenburg oblast of the Russian Federation and submitted to the Federal Center for Animal Health (FGBI “ARRIAH,” Vladimir, Russia) for laboratory testing on 24 December 2021 (Figure 2).

The territory of the village of Karagach, Belyaevsky district, Orenburg region was previously included in the free zone without FMD vaccination without official recognition by the OIE. After studying the clinical lesions of the oral mucosa in the cattle, the probable timing of the animal disease was provisionally determined, namely, the period from 10 to 22 December 2021.

To curb the outbreak, the following control measures were taken in Russia: disinfection, quarantine, the destruction of clinically ill animals, the quarantine and movement control of animals from around the outbreaks was imposed, surveillance in the threatened zone, ring emergency vaccination in response to the outbreak in the infected and risk zones, control of the viral reservoir in wild fauna, the official destruction of animal products, and the official disposal of carcasses and byproducts. To ease the implementation of control measures, the area was zoned into an outbreak zone (the location of the virus, transmission factors), an infected zone (the area within 5 km of infected animals), a risk zone (a zone within 30 km of the infected zone), and a surveillance zone (an area within 10 km of the risk zone).

In 2021, Mongolia reported a total of 94 outbreaks in the following aimags (regions): Bulgan—9, Hovd—8, Dornod—5, Övörhangay—8, Dundgovi—11, Töv—13, Bayan-Ölgiy—2, Darhan-Uul—1, Bayanhongor—9, Govi-Altay—9, Uvs—1, Hentiy—7, Hövsgöl—1, Arhangay—7, Ömnögovi—1, Selenge—1, Dzavhan—1. FMD with clinical symptoms was observed among cattle, sheep, and goats. In addition, 30 wild Mongolian antelopes (*Procapra gutturosa*) displayed FMD clinical signs and mortality cases (https://wahis.woah.org/#/in-review/3800?reportId=155562&fromPage=event-dashboard-url; accessed on 1 March 2022).

In February 2022, three samples of an aphthous suspension were collected from FMD-infected cattle from outbreaks in the Mongolian aimags (regions) of Sukhbaatar, Khentii, and Khovd in September–October 2021 [30].

The following control measures were implemented in Mongolia: ring emergency vaccination in response to the outbreak in the infected and risk zones, the quarantine and control of animal movement within the country, disinfection, and surveillance outside the infected and/or protection zone.

Two clinical samples (aphthous suspension) from Kazakhstan (in Kiykta, in the Shetsky district of the Karaganda region) collected in January 2022 indicated a FMD outbreak upon detection of O/ME-SA/Ind-2001e sublineage [31]. FMD outbreaks in Orenburgskaya oblast (Russia) and Karagandinskaya oblast (Kazakhstan) attributable to O/ME-SA/Ind-2001, were documented in regions that have had a long history of freedom (no vaccination): since 1973 in Orenburgskaya oblast, and 2007 in Karagandinskaya oblast.

Before the outbreak of foot-and-mouth disease in 2022, the Karaganda region of the Republic of Kazakhstan was a part of a zone that was free of FMD without vaccination, and was officially recognized by the OIE in 2015. According to the characteristic lesions of the mucous membrane of the oral cavity of infected animals on the day of detection, and considering the incubation period of FMD, the dates of the disease were provisionally determined as 15–27 December 2021. Samples from the affected areas, saliva, and blood were collected from two sick animals belonging to one owner and suspected of having FMD, and sent to the National Veterinary Referral Center. After the FMDV genome had been detected using an inhouse reverse-transcription polymerase chain reaction, samples were sent for confirmation to the Regional OIE Reference Laboratory for FMD (FGBI ARRIAH).

Kazakhstan reported the following undertaken measures to control the disease: disinfection, quarantine, the destruction of clinically ill and susceptible incontact animals, ring emergency vaccination zoning in response to the outbreak in infected and risk zones, movement control, and surveillance outside the infected zone.

More information regarding the number of susceptible and infected cattle, and measures taken can be found at https://wahis.woah.org, accessed on 23 October 2022, according to the reports submitted by the veterinary services.

To assist in the molecular epidemiological analysis of FMD reported in this manuscript, archived clinical samples from Russia and Mongolia were accessed: O/MOG/14/Ca/Do/2018 (Dornod, Mongolia, 2018), O/MOG/13/Ca/Kh/2018 (Khentii, Mongolia, 2018), O/MOG/13/2017 (Dornod, Mongolia, 2017), and O/Zabaikalskiy/RUS/2019 (Zabaikalskiy Kray, Russia, 2019).

The locations of the studied FMD outbreaks are shown in Figure 1.

### 2.2. Data Collection

To better understand the FMD epidemiology in the subregion, data from www.wrlfmd.org (accessed on 23 October 2022) and www.woah.org (accessed on 23 October 2022) on FMD outbreaks in Russia, Mongolia, Kazakhstan, and China were used in this study. The following data parameters were assessed: annual World Organization for Animal Health (WOAH) reports as of 2013 in Russia, Kazakhstan, China, and Mongolia; World Reference Laboratory FMD reports (the Pirbright Institute, UK) on the genotyping of Type O FMDV in the corresponding years. The locations of FMD outbreaks were retrieved from the WOAH World Animal Health Information System (WAHIS) at https://wahis.woah.org/#/home, accessed on 23 October 2022. Maps were created using the ArcGIS 10.8.1 geoinformation system (ESRI, Redlands, CA, USA). Background administrative boundaries were extracted from the ESRI Data and Maps dataset, publicly available at: https://www.arcgis.com/home/group.html?content=all&id=24838c2d95e14dd18c25e9bad55a7f82#overview, accessed on 23 October 2022.

### 2.3. RNA Extraction

RNA was extracted from a 10% suspension of ulcers using Trizol (invitrogen) following the manufacturer’s instructions. Prior to RNA extraction, tissue samples were crushed with a sterile grinder to prepare a 10% suspension in PBS buffer.

### 2.4. PCR and Sequencing

The VP1 gene was amplified and sequenced using previously described primers [32]. Sequencing was performed using the BigDye Terminator Cycle Sequencing kit and the ABI Prism 3130 automatic sequencer (Thermo Fisher Scientific, USA). Full-length VP 1 nucleotide sequences were analyzed using the BioEdit software package. MEGA7 software with the maximum-likelihood estimation and neighbor-joining methods, and 1000 bootstrap replicas were used to construct dendrograms. Each sequencing reaction was carried out in five replicates to ensure the consistency of the resulting VP1 sequences.

### 2.5. Virus Isolation

FMDV was isolated and reproduced using continuous monolayer cell cultures of Siberian ibex kidney cells (PSGK-30) and pig kidney cells from Instituto Biologico-Rim Suino-2 (IB-RS-2). The infected cell cultures (CCs) were incubated at 37 °C until CPE had been evident. To adapt the virus, no more than three passages were performed in the CCs. A virus that gave 90–100% CPE within 18–24 h was considered adapted [33].

Viral infectivity titration using a micromethod in IB-RS-2 CC was performed in 96-well culture plates on a continuous IB-RS-2 cell culture (concentration of 0.8–1.0 × 10^6^ cell/mL at 37 °C and 5% CO_2_ for 48 h). The viral titer was read using an inverted microscope considering the number of wells with evident specific CPE, calculated with the Karber method, and expressed as log10 TCD_50_/50 µL (TCD, tissue cytopathic dose).

### 2.6. Vaccine Matching

Reference sera were obtained at the FGBI ARRIAH from cattle vaccinated with FMD monovalent inactivated vaccines from the following strains belonging to ME–SA topotypes at 21–30 days after vaccination: O_1_ Manisa, O No.1734/Primorsky/2000; SEA: O No. 2102/Zabaikalsky/2010, O No. 2212/Primorskiy/2014 and O No. 2311/Zabaikalsky/2016 (O Ind-2001).

FMD viral isolates were matched with the production strain with the viral neutralization test (VNT) in IB-RS-2 CC. The titers of reference sera from cattle immunized with the vaccines on the basis of the production of homologous and heterologous FMDV strains were determined with the checkerboard titration method using five doses of the virus. The serum titer against 100 TCD_50_ was estimated with regression and expressed as log10. An r_1_ value was calculated as the reciprocal arithmetic log10 titer of reference serum against heterologous and homologous viruses, and was interpreted according to M. Rweyemamu (1984) [34]: r_1_ ≥ 0.3 indicates a close antigenic relationship among the strains, so the use of a vaccine based on this production strain is likely to confer protection against challenges with the field isolate; r_1_ < 0.3 indicates no antigenic relationship among the strains, and the production strain does not confer protection against the field isolate; r_1_ = 0.28–0.32 was the cut-off value range.

## 3. Results

### 3.1. Time Course of FMD Outbreaks

**Figure 3:**
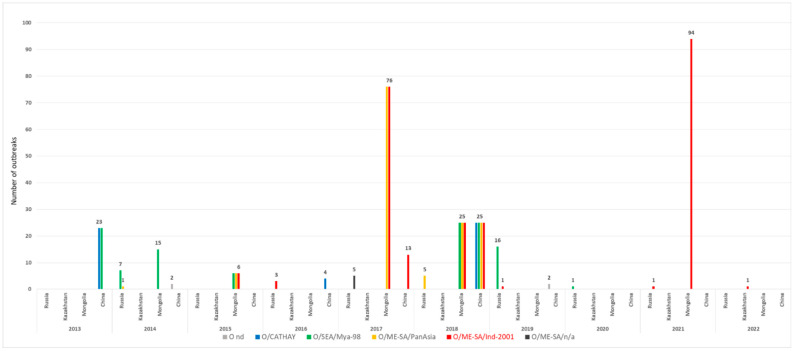
Time course of Type O foot-and-mouth disease outbreaks in Russia, Kazakhstan, and Mongolia from 2013 to 2022 (dated 1 April 2022).

Figure 3 shows the time course of O/ME-SA/Ind-2001 lineage occurrences in Russia, Kazakhstan, Mongolia, and China over a 9-year period. In 2013–2014, in China, Russia, and Mongolia, the O/Sea/Mya-98 lineage dominated. However, in 2015, both O/Sea/Mya-98 and O/ME-SA/PanAsia were prevalent, accompanied by the first incursions of O/ME-SA/Ind-2001, whereas in 2016, O/ME-SA/Ind-2001 was first reported in Russia. An increasing trend in the number of FMD outbreaks caused by O/ME-SA/Ind-2001 in Mongolia was noteworthy (from 6 cases in 2015 to 76 cases in 2017 and 94 cases in 2021).

### 3.2. VP1 Sequencing

**Figure 4:**
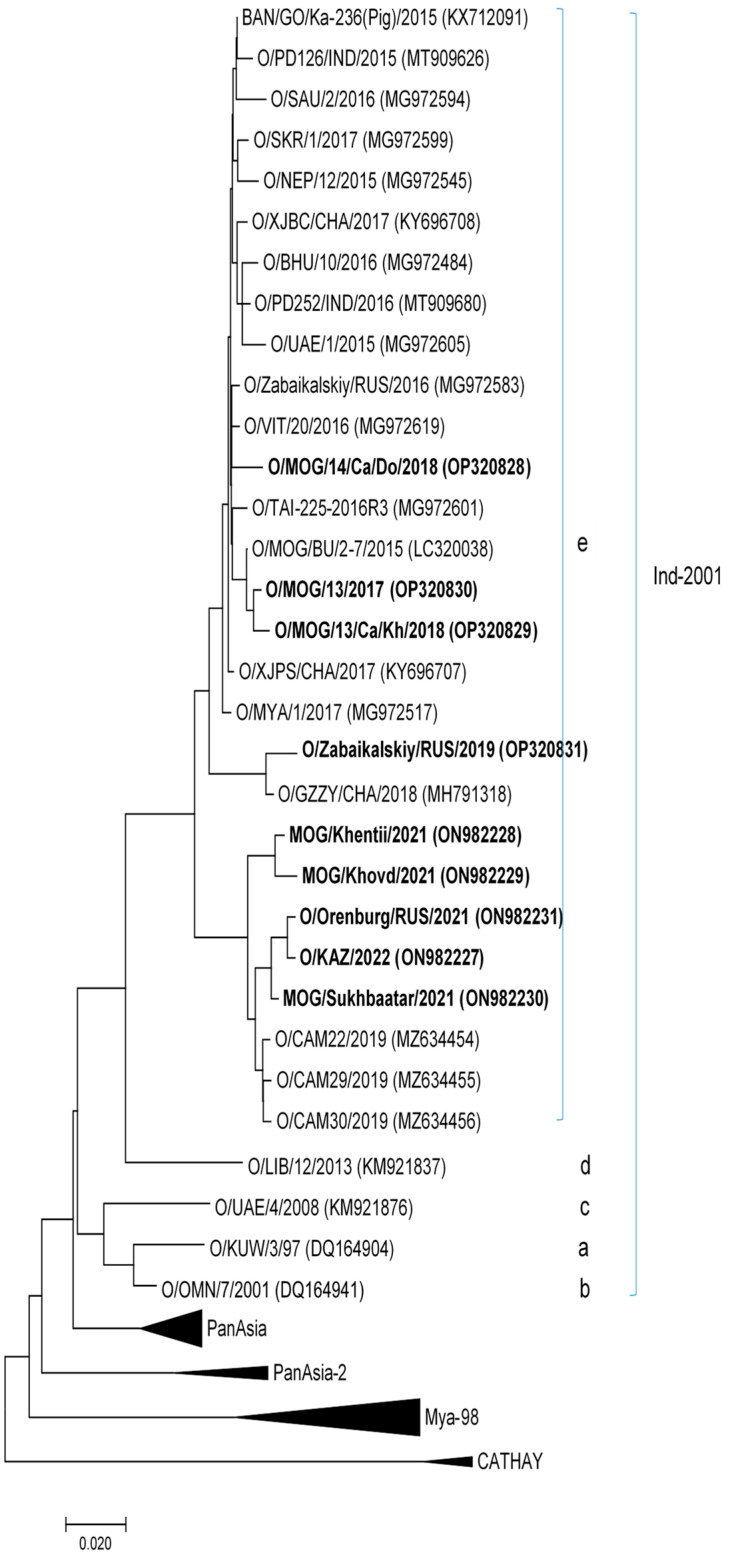
Maximum likelihood tree reflecting the phylogenetic relationship among Russian, Kazakhstan, and Mongolian foot-and-mouth disease viral isolates collected in 2021 and 2022 on the basis of full-length VP1 gene sequences. The studied isolates are in bold.

The FMD virus identified in the tested samples belonged to the ME–SA topotype (Middle East–South Asia), genetic lineage O/ME-SA/Ind-2001, according to VP1 sequencing and phylogenetic analysis (Figure 3). At the VP1 gene, the isolates from Russia, Kazakhstan, and Mongolia differed by 1.0–3.8% (Table 1). The Mongolian isolates varied by 1.0–3.3%, whereas the Russian and Kazakhstan isolates varied by only 0.8%. The Cambodian isolates from 2019 (MZ634454, MZ634455, and MZ634456) displayed the closest genetic resemblance to the isolates analyzed from Russia, Mongolia, and Kazakhstan according to our analysis of the acquired VP1 sequences against the NCBI (Figure 4).

### 3.3. Vaccine Matching

The results of antigenic variation across the FMD viral isolates recovered in Russia, Mongolia, and Kazakhstan in 2021–2022 with a VNT assay are given in Table 2.

The findings from Table 2 demonstrate that the isolates from Russia and Kazakhstan (2021–2022) antigenically differed from O_1_ Manisa (ME–SA), O No. 2102/Zabaikalsky/2010 (O/Mya-98) (r_1_ = 0.05–0.28) and bore close antigenic resemblance to O No. 2212/Primorsky/2014 (O/Mya-98) and O No. 2311/Zabaikalsky/2016 (O/Ind-2001) (r_1_ = 0.66–1.0). The Mongolian isolate was closely antigenically related to O 1734/Primorsky/2000 (r_1_ = 0.44), O No. 2212/Primorsky/2014 (r_1_ = 0.66) and O No. 2311/Zabaikalsky/2016 (r_1_ = 1.0).

On the basis of the results of antigen matching, strain No. 2311/Zabaikalsky/2016 (O/Ind-2001e) was chosen as the matching vaccine virus to curb outbreaks and prevent FMD in Russia, the Republic of Kazakhstan, and Mongolia.

**Figure 5:**
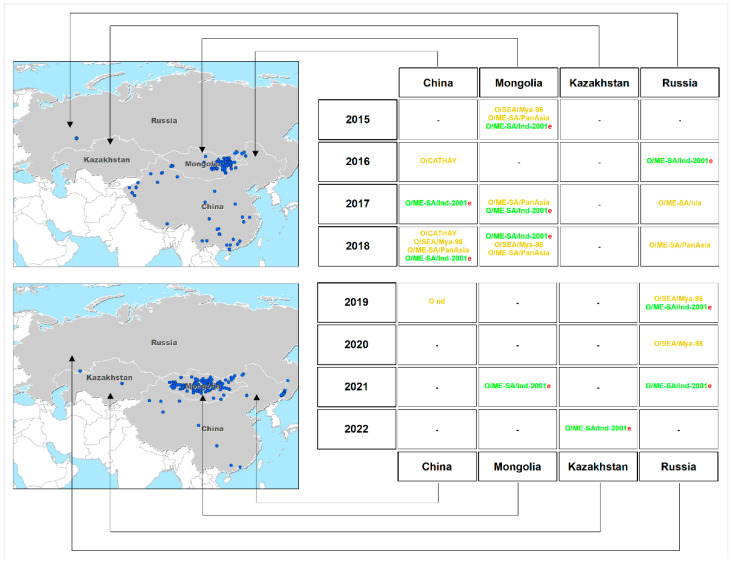
Time course of changes in the range of foot-and-mouth disease outbreaks from 2015 to 2018 and from 2019 to 2022 in Russia, Kazakhstan, Mongolia, and China.

Figure 5 demonstrates that the O/ME-SA/Ind-2001e sublineage has been present in the region for the last eight years, along with O/SEA/Mya-98 transient circulation from 2019 to 2022. In 2019, the range of outbreaks gradually expanded toward East China and the Russian Far East.

An increasing frequency of FMD outbreaks due to the O/ME-SA/Ind-2001e sublineage in Mongolia and spillovers into free regions of Russia and Kazakhstan has been observed since 2021. The representative progeny of field strains of the O/ME-SA/Ind-2001e lineage is a potent vaccine candidate against the currently circulating viral variant pool.

## 4. Discussion

This is the first study looking into genetic variation in FMDV circulating across a few countries in the Northern Hemisphere. The FMDV O/ME-SA/Ind-2001 lineage has pandemic potential, and now represents an important challenge to veterinary services and farmers.

By 2021–2022, the FMDV O/ME-SA/Ind-2001 lineage rapidly spread over a long distance in a northward direction, and caused outbreaks in Kazakhstan, Mongolia, and Russia (Figure 4). The sequence analysis carried out herein demonstrates a high level of genetic relatedness across the studied isolates that shared 1–4% differences, whereas isolates from Russia were closer to those from Kazakhstan than those from Mongolia (Table 1). Considering the average distance between the outbreaks in Russia and Kazakhstan, it is evident that O/KAZ/2022 and O/Orenburg/RUS/2021 were the closest. Interestingly, the Mongolian isolates were more genetically related to the Cambodian isolates identified in 2019 (Figure 4). This suggests that the 2021 outbreaks of FMD in Mongolia were not a continuation of the 2017–2018 epizootic, but were brought about by a novel incursion of the FMD ME–SA/Ind–2001 lineage into this country. This virus was likely introduced from the territory of the People’s Republic of China, like in all previous events, because of the wide shared border and transboundary nature of FMDV. Regrettably, there are no nucleotide sequences of Chinese isolates from 2019–2021 in the GenBank database. However, the data provided by the Chinese representative at the annual meetings of the FMD Reference Laboratory Network 2017–2021 show that O/ME-SA/Ind-2001 is already circulating in China [35].

In Russia, FMD outbreaks caused by O/ME-SA/Ind-2001 were first reported in 2016 in the Zabaikalsky Krai. That viral lineage was used to prepare a vaccine for emergency vaccination against the circulating lineage in both Russia and the neighboring countries. The reintroduction of this genetic lineage also occurred in the Zabaikalsky Krai in 2019 (Figure 4). Surprisingly, in December 2021, an FMD outbreak caused by the O/ME-SA/Ind-2001 virus was reported in the Orenburg oblast of the Russian Federation (Karagach village, Belyaevsky Raion) that, like all regions of Russia bordering Kazakhstan, had been free from FMD for several previous decades, and had not practiced vaccination for the previous two years (Figure 3). The genetic relationship (more than 99% VP1 similarity) of the FMDV O/Orenburg/2021/Rus and O/KAZ/2022 isolates, a short interval of time between the outbreaks, and the geographical location of the Russian FMD outbreak (19 km away from the state border with Kazakhstan) suggest a concurrent O/ME-SA/Ind-2001 FMDV spillover into both countries. This argues for a need to strengthen FMD surveillance using laboratory diagnostic methods in the population of wild migrating cloven-hoofed animals, which may be asymptomatic viral carriers that do not always manifest clinical signs when infected [36].

In Mongolia, FMDV O/ME-SA/Ind-2001 was first identified in March 2015 in the westernmost aimag of Bayan-Ölgii. The virus of this lineage was again detected in 2017 at the Sukhbaatar aimag. In 2018, FMDV O/ME-SA/Ind-2001 caused outbreaks in the aimags of Dornod, Sukhbaatar, and Khentii. From 2019 to 2020, Mongolia reported no FMD outbreaks. However, in 2021, multiple FMD outbreaks caused by the O/ME-SA/Ind-2001 virus were registered affecting 19 of the 21 aimags of the country; the virus was also responsible for the outbreak and deaths of wild Mongolian gazelles (Procapra gutturosa) [35]. Interestingly, antibodies to structural (Type O) and nonstructural FMDV proteins were detected in the population of Mongolian gazelles (Procapra gutturosa) migrating from Mongolia, pointing to the role of wildlife in transmitting FMDV [37].

The detection of the O/ME-SA/Ind-2001 genetic lineage virus in Kazakhstan, Russia, and Mongolia in 2021–2022 implies that the panzootic is escalating and expanding to new territories. The selection of a strain for the manufacture of vaccines effectively protecting animals is necessary in order to avoid the disease caused by the circulating the O/ME-SA/Ind-2001e sublineage, since this must be based on matching a representative field isolate from outbreaks in the region [37].

Several sociological and economic factors should be considered as contributing factors for the outbreaks of FMD. The Bayan-Ulgiy aimag is a territory of compact residence of Kazakhs on the territory of Mongolia, and its citizens constantly communicate closely with their relatives who moved to Kazakhstan after the break-up of the Soviet Union. According to the Mongolian database, 40,000 Mongolian citizens received Kazakhstani citizenship in the past 20 years (1995–2015). Therefore, the transmission of the infection via various routes is possible (transport, food, clothing, etc.).

In this study, we carried out a two-dimensional VNT utilizing reference monovalent bovine sera in order to compare the O/ME-SA/Ind-2001 virus and the strains of the existing FMD vaccine. The study revealed that FMD viral isolates found in Russia, Mongolia, and Kazakhstan in 2021–2022 were antigenically different from strains O_1_ Manisa (ME–SA), O No. 2102/Zabaikalskiy/2010 (O/Mya–98), and similar to strains O No. 2212/Primorskiy/2014 (O/Mya–98) and O No. 2311/Zabaikalskiy/2016 (O/Ind–2001) that, if formulated into FMD vaccines, could confer effective protection of animals from the FMDV O/ME-SA/Ind-2001 lineage (Table 1). The presented data allow for the use of strains O No. 2212/Primorskiy/2014 (O/Mya-98) and O No. 2311/Zabaikalskiy/2016 (O/Ind-2001) for the manufacture of monovalent FMD vaccines or their formulation into polyvalent vaccines for the prevention of foot-and-mouth disease in this region.

In conclusion, FMDV isolates that spread in Russia, Kazakhstan, and Mongolia in 2021–2022 were genetically analyzed as part of this study, and the results showed that these isolates belonged to the O/ME-SA/Ind-2001e sublineage. A closer program for FMD surveillance in the highlighted countries is needed in light of the ongoing global transboundary movement of the O/ME-SA/Ind-2001 lineage pandemic in order to develop timely intervention strategies and vaccine preparations to match the genetic diversity of the subregional strain pool.

## Figures and Tables

**Table 1:** Nucleotide identities of the isolates in this study.

Seq->	O/Orenburg/RUS/2021	O/KAZ/2022	MOG/Sukhbaatar/2022	MOG/Khentii/2022	MOG/Khovd/2022
O/Orenburg/1/RUS/2021	ID	0.992	0.989	0.967	0.962
O/KAZ/2022	0.992	ID	0.987	0.965	0.96
MOG/Sukhbaatar/2022	0.989	0.987	ID	0.971	0.967
MOG/Khentii/2022	0.967	0.965	0.971	ID	0.99
MOG/Khovd/2022	0.962	0.96	0.967	0.99	ID

**Table 2:** Antigenic relationship (r_1_) between FMDV type O isolates and production vaccine strains.

FMDV Isolates	Postvaccination Monovalent Sera against ARRIAH Production Strains, r_1_ Value				
	O_1_ Manisa (ME–SA)	O No. 1734 /Primorsky/2000 (O/PanAsia)	O No. 2102 /Zabaikalsky/2010 (O/Mya-98)	O No. 2212 /Primorsky/2014 (O/Mya-98)	O No. 2311/ Zabaikalsky/2016 (O/Ind-2001)
O/Orenburg/2021	0.28	0.04	0.08	0.7	1.0
O/Kazakhstan/2022	0.23	0.22	0.05	0.87	1.0
O/Mongolia/2021	0.28	0.44	0.06	0.66	1.0

## Data Availability

All data applicable to this article are available in this study. The reported sequence is available in GenBank under O/MOG/14/Ca/Do/2018 OP320828, O/MOG/13/Ca/Kh/2018 OP320829, O/MOG/13/2017 OP320830, O/Zabaikalskiy/RUS/2019 OP320831, O/KAZ/2022 ON982227, MOG/Khentii/2021 ON982228, O/MOG/Khovd/2021 ON982229, MOG/Sukhbaatar/2021 ON982230, O/Orenburg/RUS/2021 ON982231.
